# Avian diversity and bird-aircraft strike problems in Bahir Dar International Airport, Bahir Dar, Ethiopia

**DOI:** 10.1186/s40850-022-00135-8

**Published:** 2022-06-28

**Authors:** Tsegaye Tefera, Dessalegn Ejigu, Nega Tassie

**Affiliations:** Department of Biology, College of Science, Bahir Dar University, P.O. Box 79, Bahir Dar, Ethiopia

**Keywords:** Aircraft, Bahir Dar, Bird strike, Shannon–Wiener diversity, Species evenness

## Abstract

**Background:**

Bahir Dar International Airport and its surrounding habitats are known for their rich avifaunal diversity, which results in bird-aircraft collisions as a fundamental problem in the area. A study on bird diversity and bird-aircraft strikes at Bahir Dar International Airport was conducted between February 2020 and August 2020. Based on its vegetation structures, the study area was classified into four habitat types namely; bushland, grassland, wetland, and modified habitats. Transect and point count methods were used to collect data on avian diversity and abundance. Questionnaire surveys, interviews, and document analysis were used to gather information about incidents and protection measures against bird-aircraft strike problems. Shannon–Wiener diversity index, Simpson’s similarity index, ANOVA, and chi-square test were used for data analysis.

**Results:**

A total of 80 avian species belonging to 15 orders and 40 families were identified in the study area. The highest species diversity (H’ = 3.59) and species evenness (E = 0.96) were recorded in modified habitats during the wet season. Relative abundance categories of birds in the study area showed that most were uncommon birds. Birds pose severe threats to aircraft in the airport and 92.3% of the respondents replied that most bird-aircraft strikes occurred early in the morning and late in the afternoon when birds remain more active. The majority (88.5%) of questionnaire participants confirmed that bird-aircraft strike incidents frequently occurred during the time of takeoff and landing of the aircraft. It is also known that on average forty bird-aircraft collisions per year happen at the airport.

**Conclusion:**

Bahir Dar International Airport is rich in its bird diversity that recalls the aviation authority to work in collaboration with different organizations to avoid bird-aircraft strike problems using different control measures without compromising the conservation of birds.

**Supplementary Information:**

The online version contains supplementary material available at 10.1186/s40850-022-00135-8.

## Background

Birds are vital components of biodiversity, and they are the best known and highly significant organisms in the natural world [1]. They play a great role as bio-indicators and biocontrol agents [2, 3]. Although birds occupy most of the earth’s surface, the majority of them are found only in particular regions and habitats [4]. Topographic diversity and variability of climate in Ethiopia contribute to its rich avian diversity [5]. Moreover, millions of migratory birds come to Ethiopia having flown all the way through the eastern flyway which makes the country one of the main corridors for migratory species. However, various anthropogenic disturbances occurring in natural habitats would affect the availability of various food items that influence on diversity, abundance, and distribution of birds [6].

Ethiopia harbors over 860 species of birds and represents 9.5% of the World’s and 39% of Africa’s avian species of which nineteen species are endemic to Ethiopia, three are rare species, fourteen other species are shared with Eritrea, and thirty-one are globally threatened [4]. Moreover, over 1230 Important Bird Areas (IBAs) have already been identified in Africa of these, 73 being in Ethiopia [1].

Different environmental variables including food, temperature, and competition have been found to influence avian species diversity and abundance [4]. Urban environments provide birds with considerable quantities of food and roosting sites [7], and airports are one of the structural features of urban environments. The natural environment and human activities inside and in the immediate vicinity of airports provide a wide variety of natural and human-made habitats for birds that offer them diverse food items, nesting and roosting sites, shelter, and other facilities [8].

Bird-aircraft strike is a major hazard to the aviation industry [9] and it is one of the serious concerns for economic and flight safety reasons [10, 11]. The first worldwide recorded fatality due to a bird-aircraft collision occurred in 1912 [11, 12] and the incidents were rare during the beginning of the aviation industry, which resulted in slight damage [12]. However, the number and frequency of bird-aircraft collisions increased significantly over the last decades due to an increase in the number of flight operations combined with increasing numbers of birds of prey and small gregarious bird species, especially during migration [1]. More than one hundred bird species have been recorded to cause worldwide bird-aircraft strike problems [13]. Generally, bird-aircraft strikes cause an annual loss of about 1.2 billion USD in the global aviation industry [7]. Ethiopian airlines annually lose more than five million birr [> 100 K USD] to maintain equipment damaged by bird-aircraft strikes [14]. For example, a flock of speckled pigeons collided with Boeing-737 in 1988 at Bahir Dar International Airport resulting in the death of more than 30 people and the complete destruction of the aircraft [15].

The occurrence of birds at the airport depends on the attractiveness of habitats within and around the airports [16]. Bird-aircraft collisions are becoming a fundamental problem, especially in areas where airports are closer to water bodies, farmlands, grasslands, and damping sites [11]. Therefore, the need for effective bird control measures at airports and their vicinity has increased through the years. It is important that airport authorities show due emphasis on protecting bird-aircraft strike problems by employing effective bird control measures that are appropriate for their situation [7, 12].

There have been many studies conducted on avian ecology in East African countries including Kenya, Uganda, and Tanzania [17]. However, very few studies were conducted in Ethiopia [18]. Bahir Dar International Airport and its surrounding areas have bird-friendly habitats where diverse species of birds exist that demand research on the extent of bird-aircraft strike problems and its controlling measures. Thus, the main objective of this study is to investigate the avian diversity and bird-aircraft strikes at Bahir Dar International Airport and recommend appropriate control measures to prevent the problem.

## Results

### Species composition

A total of 80 species of birds belonging to 15 orders and 40 families were identified at Bahir Dar International Airport. Seasonal avian diversity showed that 79 and 69 species were recorded during the wet and dry seasons, respectively, of which 68 species were common both during the wet and dry seasons. But eleven species of birds were recorded only during the wet season, while one species was recorded only during the dry season (Table 1).

**Table 1:** List of bird species recorded during wet and dry seasons in the study area and their distribution in the four study habitats

Common name	Scientific name	Family	Order	**Habitat types**				**Seasons**		
				Bushland	Grassland	Modified habitat	Wetland	dry	wet	both
Abdim's stork	*Ciconia abdimii*	Ciconiidae	Ciconiiformes		✓	✓				✓
Abyssinian ground horn bill	*Bucorvus abyssinicus*	Bucorvidae	Bucerotiformes	✓					✓	
African Sacred ibis	*Threskiornis aethiopicus*	Threskiornithidae	Pelecaniformes				✓			✓
African black duck	*Anas sparsa*	Anatidae	Anseriformes				✓			✓
African black-headed oriole	*Oriolus larvatus*	Oriolidae	Passeriformes	✓						✓
African darter	*Anhinga rufa*	Anhingidae	Suliformes				✓			✓
African fish eagle	*Haliaeetus vocifer*	Accipitridae	Accipitriformes				✓			✓
African grey hornbill	*Lophoceros nasutus*	Bucerotidae	Bucerotiformes	✓						✓
African hoopoe	*Upupa africana*	Upupidae	Bucerotiformes		✓	✓				✓
African jacana	*Actophilornis africanus*	Jacanidae	Charadriiformes				✓			✓
African mourning dove	*Streptopelia decipiens*	Columbidae	Columbiformes		✓	✓				✓
African open billed stork	*Anastomus lamelligerus*	Ciconiidae	Ciconiiformes		✓		✓		✓	
African paradise Monarch	*Terpsiphone viridis*	Monarchidae	Passeriformes	✓	✓	✓				✓
African spoon bill	*Platalea alba*	Threskiornithidae	Pelecaniformes				✓			✓
African thrush	*Turdus pelios*	Turdidae	Passeriformes		✓	✓				✓
African wattled lapwing	*Vanellus senegallus*	Charadriidae	Charadriiformes		✓	✓	✓			✓
Black-billed barbet	*Lybius guifsobalito*	Lybiidae	Piciformes	✓		✓			✓	✓
Black-billed wood dove	*Turtur abyssinicus*	Columbidae	Columbiformes		✓	✓				✓
Black-billed wood hoopoe	*Phoeniculus somaliensis*	Phoeniculidae	Bucerotiformes	✓		✓				✓
Black crake	*Amaurornis flavirostra*	Rallidae	Gruiformes				✓			✓
Black-headed heron	*Areda melanocephala*	Ardeidae	Pelecaniformes		✓		✓			✓
Black-headed weaver	*Ploceus melanocephalus*	Ploceidae	Passeriformes	✓		✓				✓
Black-winged love bird	*Agapornis taranta*	Accipitridae	Accipitriformes	✓		✓				✓
Cardinal woodpecker	*Dendropicos fuscescens*	Picidae	Piciformes	✓						✓
Cattle egret	*Bubulcus ibis*	Ardeidae	Pelecaniformes		✓		✓			✓
Common bulbul	*Pycnonotus barbatus*	Pycnonotidae	Passeriformes	✓		✓				✓
Common fiscal	*Lanius collaris*	Laniidae	Musophagiformes	✓		✓				✓
Common Sand piper	*Actitis hypoleucos*	Scolopacidae	Charadriformes				✓		✓	
Dark chanting goshawk	*Melierax metabates*	Accipitridae	Accipitriformes	✓		✓				✓
Double toothed barbet	*Lybius bidentatus*	Lybiidae	Piciformes	✓		✓			✓	
Eastern Grey plantain eater	*Crinifer zonurus*	Musophagidae	Musophagiformes	✓		✓				✓
Egyptian goose	*Alopochen aegyptiaca*	Anatidae	Anseriformes		✓		✓			✓
Giant kingfisher	*Megaceryle maxima*	Alcedinidae	Coraciiformes	✓						✓
Glossy ibis	*Plegadis falcinellus*	Threskiornithidae	Pelecaniformes				✓			✓
Great white egret	*Ardea alba*	Ardeidae	Pelecaniformes		✓		✓			✓
Great white pelican	*Pelecanus onocrotalus*	Pelecanidae	Pelecaniformes				✓			✓
Greater blue-eared starling	*Lamprotornis chalybaeus*	Sturnidae	Passeriformes	✓	✓	✓				✓
Grey woodpecker	*Dendropicos goertae*	Picidae	Piciformes	✓		✓				✓
Grey-headed kingfisher	*Halcyon leucocephala*	Alcedinidae	Coraciiformes	✓				✓		
Northern Grey headed sparrow	*Passer griseus*	Passeridae	Passeriformes	✓	✓	✓				✓
Grey heron	*Ardea cinerea*	Ardeidae	Pelecaniformes				✓			✓
Hadada ibis	*Bostrychia hagedash*	Threskiornithidae	Pelecaniformes		✓	✓	✓			✓
Hamerkop	*Scopus umbretta*	Scopidae	Pelecaniformes				✓			✓
Helmeted Guineafowl	*Numida meleagris*	Numididae	Galliformes	✓	✓					✓
Hooded vulture	*Necrosyrtes monachus*	Accipitridae	Accipitriformes		✓	✓				✓
Knob-billed duck	*Sarkidiornis melanotos*	Anatidae	Anseriformes				✓			✓
Laughing dove	*Spilopelia senegalensis*	Columbidae	Columbiformes	✓	✓	✓				✓
Lemon dove	*Columba larvata*	Columbidae	Columbiformes		✓	✓				✓
Little egret	*Egretta garzetta*	Ardeidae	Pelecaniformes				✓			✓
Long-crested eagle	*Lophaetus occipitalis*	Accipitridae	Accipitriformes	✓		✓				✓
Marabou stork	*Leptoptilos crumenifer*	Ciconiidae	Ciconiiformes				✓			✓
Namaqua dove	*Oena capensis*	Columbidae	Columbiformes			✓				✓
Northern black flycatcher	*Melaenornis edolioides*	Muscicapidae	Passeriformes	✓		✓			✓	
Nubian woodpecker	*Campethera nubica*	Picidae	Piciformes	✓		✓				✓
Olive thrush	*Turdus olivaceus*	Turdidae	Passeriformes	✓	✓	✓				✓
Pied crow	*Corvus albus*	Sturnidae	Passeriformes	✓	✓	✓				✓
Pin-tailed whydah	*Vidua macroura*	Viduidae	Passeriformes			✓				✓
Red-billed firefinch	*Lagonosticta senegala*	Anatidae	Anseriformes			✓				✓
Red-billed oxpecker	*Buphagus erythrorhynchus*	Buphagidae	Passeriformes		✓				✓	
Red-cheeked cordon bleu	*Uraeginthus bengalus*	Estrildidae	Passeriformes	✓					✓	
Red-eyed dove	*Streptopelia semitorquata*	Columbidae	Columbiformes		✓	✓				✓
Rueppell’s robin chat	*Cossypha semirufa*	Muscicapidae	Passeriformes			✓				✓
Ruppell’s weaver	*Ploceus galbula*	Ploceidae	Passeriformes	✓		✓				✓
Senegal thick-knee	*Burhinus senegalensis*	Burhinidae	Charadriformes				✓		✓	
Silvery-checked hornbill	*Bycanistes brevis*	Bucerotidae	Bucerotiformes	✓		✓				✓
Speckled mousebird	*Colius striatus*	Coliidae	Coliiformes	✓		✓				✓
Speckled pigeon	*Columba guinea*	Columbidae	Columbiformes	✓	✓	✓				✓
Spur-winged goose	*Plectropterus gambensis*	Anatidae	Anseriformes				✓			✓
Spur-winged lapwing	*Vanellus spinosus*	Laridae	Charadriiformes				✓			✓
Striped kingfisher	*Halcyon chelicuti*	Alcedinidae	Coraciiformes	✓		✓				✓
Swainson’s sparrow	*Passer swainsonii*	Passeridae	Passeriformes		✓					✓
Tawny eagle	*Aquila rapax*	Accipitridae	Accipitriformes	✓						✓
Tropical boubou	*Laniarius major*	Malaconotidae	Passeriformes	✓		✓				✓
Village indigobird	*Vidua chalybeate*	Viduidae	Passeriformes			✓				✓
Wattled ibis	*Bostrychia carunculata*	Threskiornithidae	Pelecaniformes		✓		✓		✓	
White-faced whistling duck	*Dendrocygna viduata*	Anatidae	Anseriforme				✓			✓
Yellow billed egret	*Ardea intermedia*	Ardeidae	Pelecaniformes				✓			✓
Yellow-billed kite	*Milvus aegyptius*	Accipitridae	Accipitriformes	✓	✓	✓				✓
Yellow-fronted canary	*Crithagra mozambica*	Fringillidae	Passeriformes	✓	✓	✓			✓	
Yellow wagtail	*Motacilla flava*	Motacillidae	Passeriformes		✓					✓

**Fig. 1:**
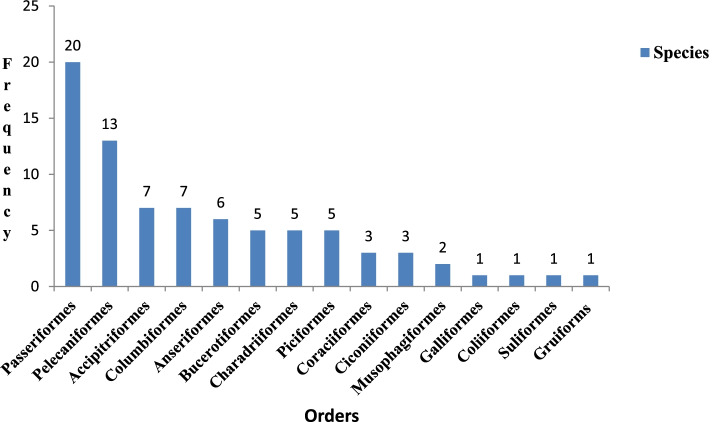
Frequencies of species within each order

The highest number of families were recorded for the order Passeriformes (14 families) followed by Charadriiformes (5 families), Pelecaniformes and Bucerotiformes (4 families each), and the lowest was recorded under the orders Anseriformes, Accipitriformes, Columbiformes, Coraciiformes, Ciconiiformes, Gruiformes, Galliformes, Coliiformes, and Suliformes (1 family each). Moreover, order Passeriformes had the highest number of species (20 species), followed by Pelecaniformes (13 species), Accipitriformes and Columbiformes (7 species each), Anseriformes (6 species), Bucerotiformes, Charadriiformes, and Piciformes (5 species each), Coraciiformes and Ciconiiformes (3 species each), Musophagiformes (2 species), and the other four orders were found to be with the lowest number of species (1 species each) (Fig. 1).

In addition, the conservation status of birds was identified using International Union for Conservation of Nature (IUCN) Red List and National Red List Data Book. Among the total 80 species of birds recorded in the study area, two species; wattled ibis (*B. carunculata*) and black-winged lovebird (*A. taranta*) were endemic to Ethiopia and Eritrea, one species namely the hooded vulture (*N. monachus*) was critically endangered, and two species i.e., tawny eagle (*A. rapax)* and Abyssinian ground hornbill *(B. abyssinicus*) were vulnerable. Out of the total species of birds recorded in the area, 14 species were migrants and 66 were found to be residents.

### Species diversity

Avian species diversity varied among the four habitat types. The highest species diversity (H´ = 3.59) was recorded in modified habitats during the wet season and the lowest (H´ = 2.78) was in grassland habitats during the dry season. The highest species evenness (E = 0.96) was obtained in modified habitats during the wet season, while the lowest (E = 0.91) was in wetland and grassland habitats during the dry season. The highest species richness (42 species) was recorded in modified habitats during the wet season and the lowest (21 species) was in grassland habitats during the dry season (Table 2).

**Table 2:** Species diversity of birds in the four study habitats

Habitat type	Seasons	No of species	No of individuals	H´	Hmax	E
Bushland	Dry	29	1016	3. 11	3.37	0.92
	Wet	35	1184	3.34	3.55	0.94
Grassland	Dry	21	947	2.78	3.04	0.91
	Wet	29	1073	3.22	3.36	0.95
Modified habitat	Dry	31	1145	3.20	3.43	0.93
	Wet	42	1720	3.59	3.73	0.96
Wetland	Dry	25	1239	2.94	3.22	0.91
	Wet	28	1628	3.14	3.33	0.94

Key: H’ = Shannon-Weiner deiversity index, Hmax = lns, E = Shannon’s equitability index

The overall avian diversity in the whole study area showed that relatively more species diversity (H´ = 4.14) and evenness (E = 0.94) were recorded during the wet season compared to the dry season (Table 3).

**Table 3:** Specie diversity of birds in the whole study area during dry and wet seasons

Seasons	No of species	No of individuals	H´	Hmax	E
Dry	69	4347	3. 89	4.23	0.92
Wet	79	5605	4.14	4.36	0.94

### Abundance of birds

There were 5605 and 4347 individual birds recorded in the study area during the wet and dry seasons, respectively, and this showed that relatively the highest number of individual birds were obtained during the wet season. However, the overall abundance of birds in the area did not show a significant difference between the two seasons (F (1,146) = 0.70; *p* > 0.05). During the wet season, the abundance of birds showed significant differences among the four habitat types (F (3,130) = 4.44; *P* < 0.05). Birds’ abundance as a function of season and habitat type has also confirmed that season and habitat are not related (χ^2^(4) = 2, df = 1; *p* = 0.157). And the relative abundance of avian species did not show a significant difference between the wet and dry seasons (F (1,146) = 0.86; *P* > 0.05).

In addition, during the dry season, speckled pigeon (*C. guinea*) with 365 individuals was relatively the most abundant species in the study area followed by greater blue-eared glossy starling (*L. chalybaeus*) with 267 individuals, and red-eyed dove (*S. semitoruquata*) with 218 individuals. During the wet season, speckled pigeon with 281 individuals was also relatively the most abundant species followed by black-headed weaver (*P. melanocephalus*) with 252 individuals, and great white pelican (*P. onocrotalus*) with 202 individuals. Thus, speckled pigeon was found to be the most abundant species during the two seasons. On average, relatively the most abundant avian species during the dry and the wet seasons in their decreasing order were speckled pigeon (*n* = 323), greater blue-eared glossy starling (*n* = 227), black-headed weaver (*n* = 225), red-eyed dove (*n* = 191), and great white pelican (*n* = 189) (Supplemental Tables 2 and 3).

### Species similarity

Simpson’s similarity index (SI) of avian species in the four study habitats showed that the highest (SI = 0.74) and the lowest (SI = 0.54) species similarity were recorded between bushland and modified habitats during the wet and dry seasons, respectively, (Table 4).

**Table 4:** Similarity of bird species among the study habitats during the wet and dry seasons

Habitats	Bushland		Grassland		Modified habitat		Wetland	
	wet	dry	wet	dry	wet	dry	wet	dry
Bushland	1	1	0.64	0.65	0.74	0.54	0.64	0.63
Grassland	-	-	1	1	0.70	0.58	0.66	0.65
Modified habitat	-	-	-	-	1	1	0.64	0.57
Wetland	-	-	-	-	-	-	1	1

### Bird-aircraft strike problems

A structured questionnaire was administered to 23(88.5%) male and 3(11.5%) female respondents. Professionally, 20(76.9%) of the respondents were bird controllers, 4(15.4%) officers, and 2(7.7%) section heads who have years of work experience in the airport.

Of the total respondents, 21(80.8%) of them confirmed that they have seen bird-aircraft collision incidents in the airport. The problem was more frequent during the summer as it was supported by 17(65.4%) of the respondents. The majority 24(92.3%) of questionnaire participants replied that most bird-aircraft strike problems occurred early in the morning and late in the afternoon.

Regarding bird-aircraft strike occurrences, the majority of respondents replied that they encountered dead birds due to collisions with aircraft. It was also known that forty bird aircraft collisions per year occurred at the airport. The majority 23(88.5%) of the study participants recalled that bird-aircraft strike incidents frequently occurred during the time of takeoff and landing of the aircraft.

Although 19 (73.1%) of the respondents replied that the speckled pigeon (*n* = 323) was the most problematic avian species frequently causing bird-aircraft strike problems at the airport, other species such as marabou stork (*n* = 44), yellow-billed kite (*n* = 37), Egyptian goose (*n* = 80), and tawny eagle *(n* = 23) were also usually involved in the bird-aircraft strike incidents at the same airport. Besides birds, other wildlife species such as hyena (*Crocuta crocuta*), Ethiopian hare (*Lepus starcki*), and common duiker (*Sylvicapra grimmia*) were also involved in wildlife-aircraft strike problems.

There are different controlling methods used by the airport office to prevent bird-aircraft strikes. These include selective removal of trees, mowing of grasses, surveying of birds and other animals using vehicles, removing birds’ nests around the airport, draining ditches of water, using sounds of guns for large flocks of birds, discouraging birds using whips, removing dead bodies and other wastes, and establishing strong security fences along the runway to prevent large land-dwelling animals. The majority (73.1%) of the respondents confirmed that most of the strike controlling measures include expelling birds and other wildlife away from the airport area during takeoff and landing of aircraft.

## Discussion

A total of 80 avian species were identified in Bahir Dar International Airport during the study period. This showed that this airport is rich in its avifauna diversity compared to similar airports in the other parts of the country such as Mekele International Airport which harbors 68 avian species [19]. This might be due to differences in resource availability and proximity of the airport to Lake Tana, one of the five Biosphere Reserves in Ethiopia [20].

Most species of birds in the Bahir Dar International Airport are available in the area throughout the year. However, there are some avian species that are observed only during the wet season. This might be due to variations in food availability and weather conditions [21]. The highest number of avian species was recorded under the order Passeriformes, which is in line with similar research findings in other parts of Ethiopia [22–24]. The modified habitat harbors relatively the highest number of species throughout the year, which is also supported by the findings of [23] that emphasize the presence of diversified microhabitats in modified habitat contributes to this result. Moreover, the food shifting behavior of birds when food is scarce during the dry season would also result in an increase in avian diversity in the modified habitat.

The lowest avian species diversity was recorded in grassland habitats during the dry season. This could be associated with scarcity of food sources and the occurrence of various anthropogenic disturbances in grassland habitat. This is also in line with the findings of [25] and [26] who claimed that anthropogenic activities including overgrazing, habitat degradation, and habitat fragmentation eventually cause migration and local extinction of birds. According to [27], avian species abundance is directly or indirectly affected by spatial variation and the degree of anthropogenic activities. Furthermore, [21] reported that the distinct seasonality of rainfall and variations in the availability of food sources contribute to variations in the abundance of avian species between the wet and dry seasons.

The highest avian species evenness was recorded in modified habitats during the wet season, while the lowest was in wetland and grassland habitats during the dry season. This indicated that in modified habitat successful avian species equally forage the available resources and this contributes to relatively higher avian species evenness in this type of habitat. In contrast in wetland and grassland habitats, feeding guild-specific bird species out-compete the available resources and they become dominant in the utilization of the available resources, which contributes to reducing species evenness. The differences in resource competition, breeding nature, foraging habit, and niche specialization among the distinct species of birds in each habitat result in fewer species evenness [28–30].

The highest and the lowest number of birds were recorded in modified habitats and grassland habitats during the wet and dry seasons, respectively. This difference might be associated with variations in resource availability among the different habitats. Moreover, the difference in abundance of birds between the modified and grassland habitats could also result in variation in the degree of anthropogenic disturbances between the two habitats. This result is similar to the findings of [21] who reported that variations in the abundance of birds are determined by food availability and breeding sites.

Relative abundance categories of birds in the study area showed that most of them were uncommon birds since out of the total 80 avian species identified in the study area, only 12 species were frequent, but all other 68 species were uncommon birds. This might be associated with better niche specialization of the uncommon birds in the area. Consistent with this result, [31] described that the presence of uncommon birds in a certain area might be due to the breeding nature, large home range, and niche requirement of the species. The result of this study is also in line with the findings of [32] who reported that the majority of birds in Bole International Airport were found to be uncommon birds.

The highest and the lowest avian species  similarity were recorded between the bushland and modified habitats during the wet and dry seasons, respectively. The similarity of avian communities between two different habitats might be due to their geographical proximity, similar ecology, and similar extent of disturbances in such habitats. The lowest avian community similarity between different habitats could be due to habitat-specific differences in foraging adaption and the response of birds to different anthropogenic disturbances. The result of this study is in line with the findings of [33] who described that the similarity of avian species composition between different habitats indicates a tendency for similar habitats to have similar species composition. Hence, in the present study, the highest and the lowest avian community similarities between bushland and modified habitats during the dry season might be influenced by differences in seasonal variation in the two habitats.

The questionnaire and interview results about bird-aircraft strike problems in Bahir Dar International Airport indicated that the majority of the respondents have observed birds die from these strikes. It is also reported that on average forty bird-aircraft strikes occurred annually in the airport. Although the strikes did not cause considerable damage to the aircraft, a substantial number of birds were found dead from these strike incidents. A study conducted by [18] reported that thirty-six bird-aircraft strikes per year occurred in Bole International Airport. To minimize bird-aircraft strikes, the aviation authority should use different bird-controlling measures in places where the competition for space between airports and birds is the strongest [34].

The respondents of this study described that most bird-aircraft strikes in Bahir Dar International Airport took place during takeoff and landing especially early in the morning and late in the afternoon. There seems to be an association between the time of the strike and the behavior of birds. This could be due to the occurrence of more aircraft traffic density and higher activity of birds during these times of the day. Similarly, [35] reported that 93% of the collisions occurred during the takeoff run, in the first phase of ascend, and in the final stage of landing. This result is also supported by other research findings [36] which described that bird-aircraft strikes are most frequently occurring in the morning and in the evening when birds are more active in foraging.

The respondents of this study also reported that most bird-aircraft strikes in Bair Dar International Airport occurred during the summer season. Besides the foggy weather condition, food and other resources are more abundant during summer which results in increasing the size of the local bird population with a subsequent increase in collision frequencies in the airport. This is in line with the findings of [37] who described that the frequency and distribution of bird-aircraft strikes had peaks that coincided with the period of migration of birds. Like other airports [9], bird-aircraft strikes in Bahir Dar International Airport are a regular threat to flight operation. Birds pose a real threat to flight safety although most collisions do not end in catastrophes [12].

In Bahir Dar International Airport the most catastrophic and fatal bird strike incident that claimed the lives of thirty-five people occurred in 1988. The majority of birds that are known to cause aircraft strikes during landing and takeoff in the airport include speckled pigeons, marabou stork, yellow-billed kite, Egyptian goose, and a tawny eagle in their decreasing order of causing the strikes. Most bird-aircraft strikes in Bole International Airport were also caused by the most abundant bird species, the pigeons [18] as higher abundance is positively correlated with the number of strikes. One of the main factors for the increase in the frequency of bird-aircraft strikes is increasing the number of birds in the area [38, 39]. Similarly, in Bahir Dar International Airport it is the speckled pigeon with relatively the highest individual abundance that causes frequent bird-aircraft strike problems. Moreover, other avian species such as the greater blue eared glossy starling, black-headed weaver, red-eyed dove, and great white pelican are often involved in bird-aircraft strike incidents. On the contrary, species with lower population sizes such as marabou stork, yellow-billed kite, Egyptian goose, and tawny eagle are also considered to be problematic species regarding bird-aircraft strike incidents in this airport. This showed that the abundance of birds in the area is not the only factor that has been correlated with bird-aircraft strike problems, but the behavioral activity of each bird might also play a significant role in such incidents.

Besides birds, land-dwelling animals such as hyenas and Ethiopian hares were also reported to pose strikes in the airport during takeoff and landing of the aircraft. This result is similar to the findings of [39] who described that large ground-dwelling animals can cause problems to aircraft operations and aircraft movements.

The main controlling measures for bird-aircraft strikes used by Bahir Dar International Airport include expelling birds away from the landing and takeoff areas. Thus, the aviation authority office needs to use varieties of bird-controlling measures including different scarring devices and habitat management techniques to discourage birds and other wildlife species from the airport vicinity.

## Conclusion 

Bahir Dar International Airport is known for its rich avifaunal diversity with a relatively high population size for each species. This diverse avian species community is because of the availability of different habitats and sufficient resources. More importantly, the proximity of Bahir International Airport to Lake Tana and associated wetlands enables the area to harbor relatively high avian species diversity. However, habitat changes due to various anthropogenic activities notably livestock grazing, and expansion of farmlands have negatively affected the diversity and abundance of birds in the area.

The findings of the present study revealed that most bird-aircraft strikes occurred early in the morning and late in the afternoon when birds remain more active. Hence, to minimize bird collisions with aircraft, the aviation authority should revise the flight schedules and try to make less traffic load early in the morning and late in the afternoon. It is also better to develop appropriate habitat management options which attract a lower number of birds into the airport. Moreover, the aviation authority in collaboration with different organizations should design and implement comprehensive protective strategies including visual, tactile, auditory, and chemical repellents to control the population of birds in the airport and avoid bird-aircraft strikes. Moreover, the office should also use appropriate risk assessment methods, especially for those birds which cause the greatest risk, and target them to control and avoid the strike problems.

## Methods

### Description of the study area

**Fig. 2:**
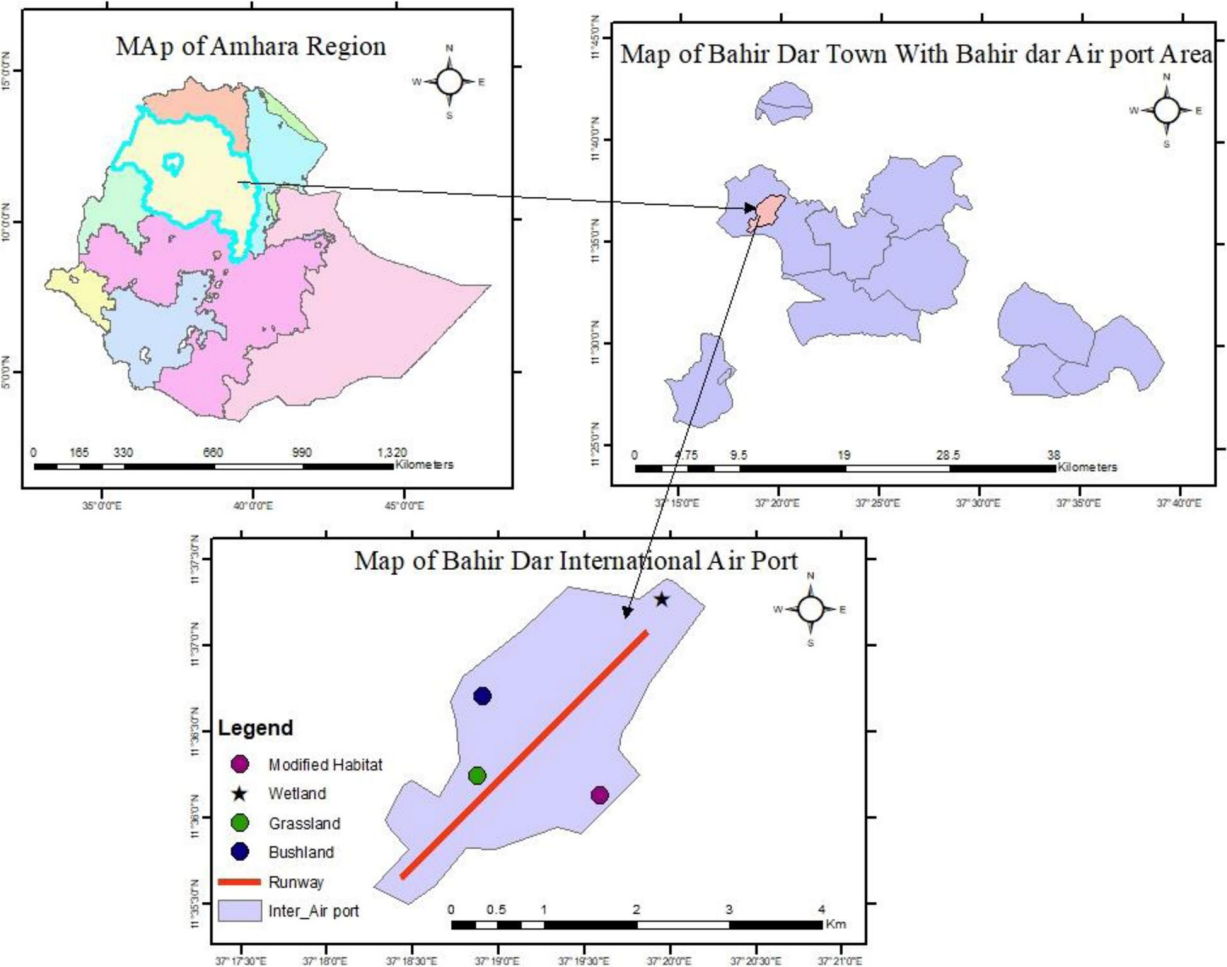
Location map of the study area

Bahir Dar International Airport, established in 1954, is one of the International Airports in Ethiopia located 8 km to the northwest of Bahir Dar City, the capital of Amhara National Regional State. It is geographically located at 11°36′30″N latitude and 37°19′30″E longitude at an elevation of 1821 m a.s.l (Fig. 2). Its main runway length and width are 3100 m and 45 m, respectively. The airport and its surrounding habitats are dominated by grassland, bushland, wetland, and modified habitats. There are a lot of tourist attraction sites around the airport including ancient monasteries and churches on the Islands of Lake Tana. Furthermore, the airport’s scenery with the Lake Tana and the beautiful city Bahir Dar create great pleasure for the travelers.

Ten years of rainfall and temperature data of the study area showed that the highest average monthly rainfall was 391.92 mm recorded during July and the lowest was 1.02 mm during January and the average monthly minimum and maximum temperatures were 6.46°c and 31.87°c recorded during January and April, respectively [40].

## Sampling design

The study area was classified into four habitat types and the sampling unit within each habitat type was determined based on vegetation type, structure, and area coverage. As a result, it is classified into bushland, grassland, wetland, and modified habitats. Modified habitat in this study is described as a habitat where human activities change their original status including aircraft runways, adjacent habitats inside the airport premises, and farmlands and grazing lands outside but closer to the airport. Among the four habitat types, three habitats namely bushland, grassland, and modified habitats are found inside the airport, while the wetlands and additionally modified habitats are located immediately outside the airport directly on the way to Lake Tana. Line transect and point count methods were used for studying the diversity and relative abundance of birds [41] in and around the airport.

The line transect method was used in wetland, grassland, and modified habitats since the areas are open, while the point count method was applied in relatively dense bushland habitats [42]. Blocks and sampling plots were established for transect and point count methods, respectively. The number of blocks and sampling plots were determined according to the size and type of vegetation cover of the study habitats. The average length and width of the transects were 200 m and 50 m, respectively. Transects were separated from each other by 100 m, and a total of 10 sample blocks (each comprising five transect lines) and eleven sample plots (each comprising five sampling points) were used.

Data on bird-aircraft strike incidents and methods of its control were assessed using questionnaire, interviews, and document analysis techniques. As a result, twenty-six respondents (twenty-three males and three females) among 100 officers and field workers were selected using the purposive sampling method. The selection of the respondents was made on the basis of the relevance of their jobs to bird strike control activities in the airport area.

## Data collection

The point count method was used to collect data in the bushland habitat, and transects were used in the wetland, grassland, and modified habitats. During the point count method, suitable sites were selected and birds were identified and counted from a fixed position within a 25 m radius for a specific period of 10 min at every point. All birds seen and heard within this 25 m radius were recorded. To minimize the disturbance during counting, a waiting period of 5 min prior to counting was applied.

Using the transect count method, birds were counted by walking at 2 km per hour and at a uniform pace throughout the whole transects. However, sometimes the speed of walking on the routes was determined by the number of birds present and the extent of difficulties in recording them.

Dry season data were collected from February to April 2020 and data for the wet season were collected from June to August 2020. Census data for the dry and wet seasons were collected twice a day when most birds are active early in the morning (6:30–9:00 a.m.) and late in the afternoon (4:00–6:30 p.m.) for five days per month with a total of 150 survey hours during the whole study period.

Field observations were made to identify birds at the species level using binoculars with a magnification power of 10 and an objective lens diameter of 50 (10 × 50). Species identifications were carried out using an appropriate field guidebook [43] and photographs were taken for further identification of birds. Movement patterns of birds that usually cross the runway were recorded to evaluate the problems of birds to aircraft strikes. The time of the day when the bird flight was the highest and activities performed by the birds such as flight direction and flock size were also recorded.

To assess the extent of bird-aircraft strike problems in Bahir Dar International Airport, questionnaire surveys were administered to 23(88.5%) male and 3(11.5%) female respondents. Structured interviews were employed to collect additional data to assess the status of bird-aircraft strike problems and their control measures applied in the airport. Secondary data were also obtained from the aviation authority office, Bahir Dar branch, to access previous information about bird-aircraft strikes in the airport.

## Data analysis

Association of birds and seasons with habitat types were analyzed using the chi-square test, and one-way analysis of variance (ANOVA) was used to check the mean abundance of species differences among the four habitat types and between seasons. Moreover, avian species diversities in each habitat type were calculated using Shannon–Wiener diversity (H'), and evenness (E) indices [4].

Shannon Wiener diversity index is calculated as:

H' =—Σ Pi × Ln (Pi) where,

H' = Shannon–Wiener diversity index.

Pi = the proportion of each species in the sample.

Ln (Pi) = natural logarithm of this proportion.

Species evenness is by Shannon’s equitability index (E) which is calculated by:

**E=** $$\frac{\mathbf{H}\boldsymbol{^{\prime}}}{\mathbf{H}\mathbf{m}\mathbf{a}\mathbf{x}}$$ where,

E = Shannon–Wiener evenness index.

H' = Shannon–Wiener diversity index.

Hmax = lns.

Ln = Log normal.

S = Total number of species.

Simpson’s similarity index (SI) was also used to evaluate the similarity of species between two different habitats in both seasons using the following formula:

SI = 2C/A + B where,

SI = Simpson’s similarity index,

A = number of species that occur in habitat ‘A’.

B = Number of species that occur in habitat ‘B’.

C = Number of common species that occur in both habitat ‘A’ and ‘B’.

The relative abundance of bird species in each habitat was calculated by:

Relative abundance=$\frac{\mathrm{n}}{\mathrm{N}}\times 100$ where,

*n* = Number of individual species.

N = the total number of individuals of all species.

Relative abundance values were used to ordinarily categorize each species under the following five abundance categories [42] (Table 5).

**Table 5:** Relative abundance score categories

Relative abundance	Relative abundance score	Abundance category
< 0.1	1	Rare
0.1–2.0	2	Uncommon
2.1–10.0	3	Frequent
10.1–40.0	4	Common
> 40	5	Abundant

## Supplementary Information

**Additional file 1: Suplemetal Table 1.** Bird order, family, genera,species, status and lifestyle of birds in the study area. **Suplementary Table 2.** Relative abundance of birds in dry season. **Suplemetal Table 3.** Relative abundance of birds during wet season.

## Data Availability

All data generated and analyzed during this manuscript preparation are available on the hands of the corresponding author.

## Abbreviations

ANOVA: Analysis of Variance
IBAs: Important Bird Areas
IUCN: International Union for Conservation of Nature
