# Supplementary material for: Avian diversity and bird-aircraft strike problems in Bahir Dar International Airport, Bahir Dar, Ethiopia
Source: BMC Zool. 2022 Jun 28;7:36. doi: 10.1186/s40850-022-00135-8 (PMC10127315; doi:10.1186/s40850-022-00135-8)
Supplement: Supplementary file 1 — Additional file 1: Suplemetal Table 1. Bird order, family, genera,species, status and lifestyle of birds in the study area. Suplementary Table 2. Relative abundance of birds in dry season. Suplemetal Table 3. Relative abundance of birds during wet season. [file 40850_2022_135_MOESM1_ESM.docx]

**Supplementary data**

**Suplemetal Table 1: Bird order, family, genera,species, status and lifestyle of birds in the study area**

| Order | Family | Common name | Scientific name | IUCN status | Lifestyle |
| --- | --- | --- | --- | --- | --- |
| Ciconiiformes | Ciconiidae | Abdim's stork | *Ciconia abdimii* | LC | Migrant |
| Bucerotiformes | Bucorvidae | Abyssinian ground horn bill | *Bucorvus abyssinicus* | VU | Resident |
| Pelecaniformes | Threskiornithidae | Africa Sacrred ibis | *Threskiornis aethiopicus* | LC | Migrant |
| Anseriformes | Anatidae | African black duck | *Anas sparsa* | LC | Resident |
| Passeriformes | Oriolidae | African black headed oriole | *Oriolus laroatus* | LC | Resident |
| Suliformes | Anhingidae | African darter | *Anhinga rufa* | LC | Resident |
| Accipitriformes | Accipitri dae | African fish eagle | *Haliaeetus vocifer* | LC | Resident |
| Bucerotiformes | Bucerotidae | African grey horn bill | *Lophoceros nasutus* | LC | Resident |
| Bucerotiformes | Upupidae | African hoopoe | *Upupa africana* | LC | Resident |
| Charadriiformes | Jacanidae | African jacana | *Actophilornis africanus* | LC | Resident |
| Columbiformes | Columbidae | African mourning dove | *Streptopelia decipiens* | LC | Resident |
| Ciconiiformes | Ciconiidae | African open billed stork | *Anastomus lamelligerus* | LC | Resident |
| Passeriformes | Monarchidae | African paradise Monarch | *Terpsiphone viridis* | LC | Resident |
| Pelecaniformes | Threskiornithidae | African spoon bill | *Platalea alba* | LC | Migrant |
| Passeriformes | Turdidae | African thrush | *Turdus pelios* | LC | Resident |
| Charadriiformes | Charadriidae | African wattled lapwing | *Vanellus senegallus* | LC | Resident |
| Piciformes | Lybiidae | Black billed barbet | *Lybius guifsobalito* | LC | Resident |
| Columbiformes | Columbidae | Black billed wood dove | *Turtur abyssinicus* | LC | Resident |
| Bucerotiformes | Phoeniculidae | Black billed wood hoopoe | *Phoeniculus somaliensis* | LC | Resident |
| Gruiformes | Rallidae | Black crake | *Amaurornis flavirostra* | LC | Resident |
| Pelecaniformes | Ardeidae | Black headed heron | *Areda melanocephala* | LC | Resident |
| Passeriformes | Ploceidae | Black headed weaver | *Ploceus cucullatus* | LC | Resident |
| Accipitriformes | Accipitri dae | Black winged love bird | *Agapornis taranta* | LC | Resident |
| Piciformes | Picidae | Cardinal woodpecker | *Dendropicos fuscescens* | LC | Resident |
| Pelecaniformes | Ardeidae | Cattle egret | *Bubulcus ibis* | LC | Resident |
| Passeriformes | Pycnonotidae | Common bulbul | *Pycnonotus barbatus* | LC | Resident |
| Musophagiformes | Laniidae | Common fiscal | *Lanius collaris* | LC | Resident |
| Charadriformes | Scolopacidae | Common Sand piper | *Actitis hypoleucos* | LC | Migrant |
| Accipitriformes | Accipitridae | Dark chanting goshawk | *Melierax metabates* | LC | Resident |
| Piciformes | Lybiidae | Double toothed barbet | *Lybius bidentatus* | LC | Resident |
| Musophagiformes | Musophagidae | Eastern Grey plantain eater | *Crinifer zonurus* | LC | Resident |
| Anseriformes | Anatidae | Egyptian goose | *Alopochen aegyptiaca* | LC | Resident |
| Coraciiformes | Alcedinidae | Giant kingfisher | *Megaceryle maxima* | LC | Resident |
| Pelecaniformes | Threskiornithid ae | Glossy ibis | *Plegadis falcinellus* | LC | Resident |
| Pelecaniformes | Ardeidae | Great white egret | *Ardea alba* | LC | Migrant |
| Pelecaniformes | Pelecanidae | Great white pelican | *Pelecanus onocrotalus* | LC | Migrant |
| Passeriformes | Sturnidae | Greater blue-eared glossy starling | *Lamprotornis chalbeus* | LC | Resident |
| Piciformes | Picidae | Grey woodpecker | *Picus canus* | LC | Migrant |
| Passeriformes | Passeridae | Grey headed sparrow | *Passer griseus* | LC | Resident |
| Pelecaniformes | Ardeidae | Grey heron | *Ardea cinerea* | LC | Migrant |
| Pelecaniformes | Threskiornithid ae | Hadada ibis | *Bostrychia hagedash* | LC | Migrant |
| Pelecaniformes | Scopidae | Hammer kop | *Scopus umbrett* | LC | Resident |
| Galliformes | Numididae | Helmeted Guinea fowl | *Numida meleagris* | LC | Resident |
| Accipitriformes | Accipitri dae | Hooded vulture | *Necrosyrtes monachus* | C/E | Resident |
| Anseriformes | Anatidae | Knob billed duck | *Sarkidiornis melanotos* | LC | Resident |
| Columbiformes | Columbidae | Laughing dove | *Spilopelia senegalensis* | LC | Resident |
| Columbiformes | Columbidae | Lemon dove | *Columba larvata* | LC | Resident |
| Pelecaniformes | Ardeidae | Little egret | *Egretta garzetta* | LC | Resident |
| Accipitriformes | Accipitridae | Long crested eagle | *Lophaetus occipitalis* | LC | Resident |
| Ciconiiformes | Ciconiidae | Marabou stork | *Leptoptilos crumenifer* | LC | Resident |
| Columbiformes | Columbidae | Namaqua dove | *Oena capensis* | LC | Resident |
| Passeriformes | Muscicapidae | Northern black fly catcher | *Melaenornis edolioides* | LC | Resident |
| Piciformes | Picidae | Nubian woodpecker | *Campethera nubica* | LC | Resident |
| Passeriformes | Turdidae | Olive thrush | *Turdus olivaceus* | LC | Resident |
| Passeriformes | Sturnidae | Pied crow | *Corvus albus* | LC | Resident |
| Coraciiformes | Alcedinidae | Grey headed kingfisher | *Halcyon leucocephala* | LC | Migrant |
| Passeriformes | Viduidae | Pin tailed whydah | *Vidua macroura* | LC | Resident |
| Anseriformes | Anatidae | Red billed firefinch | *Anas sparsa* | LC | Resident |
| Passeriformes | Buphagidae | Red billed oxpeckier | *Buphagus erythrorhynchus* | LC | Resident |
| Passeriformes | Estrildidae | Red cheeked cordon blue | *Uraeginthus bengalus* | LC | Resident |
| Columbiformes | Columbidae | Red eyed dove | *Streptopelia semitorquata* | LC | Resident |
| Passeriformes | Muscicapidae | Rueppell’s robin chat | *Cossypha semirufa* | LC | Resident |
| Passeriformes | Ploceidae | Ruppell’s weaver | *Ploceus galbula* | LC | Resident |
| Shorebirds | Burhinidae | Senegal thick knee | *Burhinus senegalensis* | LC | Resident |
| Bucerotiformes | Bucerotidae | Silvery checked horn bill | *Bycanistes brevis* | LC | Resident |
| Coliiformes | Coliidae | Speckled mouse bird | *Colius striatus* | LC | Resident |
| Columbiformes | Columbidae | Speckled pigeon | *Columba guinea* | LC | Resident |
| Anseriformes | Anatidae | Spur winged goose | *Plectropterus gambensis* | LC | Resident |
| Charadriiformes | Laridae | Spur winged lapwing | *Vanellus spinosus* | LC | Resident |
| Coraciiformes | Alcedinidae | Striped kingfisher | *Halcyon chelicuti* | LC | Resident |
| Passeriformes | Passeridae | Swainsan’s sparrow | *Passer swainsonii* | LC | Resident |
| Accipitriformes | Accipitri dae | Tawny eagle | *Aquila rapax* | VU | Migrant |
| Passeriformes | Malaconotidae | Tropical boubou | *Laniarius major* | LC | Resident |
| Passeriformes | Viduidae | Village indigobird | *Vidua chalybeata* | LC | Resident |
| Pelecaniformes | Threskiornithidae | Wattled ibis | *Bostrychia carunculata* | LC | Resident |
| Anseriforme | Anatidae | White faced whistling duck | *Dendrocygna viduata* | LC | Migrant |
| Pelecaniformes | Ardeidae | Yellow billed egret | *Ardea intermedia* | LC | Resident |
| Accipitriformes | Accipitridae | Yellow billed kite | *Milvus aegyptius* | LC | Migrant |
| Passeriformes | Fringillidae | Yellow fronted canary | *Crithagra mozambica* | LC | Resident |
| Passeriformes | Motacillidae | Yellow wagtail | *Motacilla flava* | LC | Migrant |

**LC=least concern, VU=vulnerable, C/E =critically Endangered**

| **Suplementary Table 2: Relative abundance of birds in dry season** | | | |  |  |
| --- | --- | --- | --- | --- | --- |
| **Common name** | **Scientific name** | **No of individual species** | **Dry relative abundance** | **Abundance score** | **Abundance category** |
| Abdim's stork | Ciconia abdimii | 15 | 0.345065562 | 2 | uncommon |
| Africa Sacrred ibis | Threskiornis aethiopicus | 94 | 2.162410858 | 3 | frequent |
| African black duck | Anas sparsa | 65 | 1.495284104 | 2 | uncommon |
| African black headed oriole | Oriolus laroatus | 50 | 1.150218542 | 2 | uncommon |
| African darter | Anhinga rufa | 29 | 0.667126754 | 2 | uncommon |
| African fish eagle | Haliaeetus vocifer | 19 | 0.437083046 | 2 | uncommon |
| African grey horn bill | Lophoceros nasutus | 19 | 0.437083046 | 2 | uncommon |
| African hoopoe | Upupa africana | 28 | 0.644122383 | 2 | uncommon |
| African jacana | Actophilornis africanus | 50 | 1.150218542 | 2 | uncommon |
| African mourning dove | Streptopelia decipiens | 48 | 1.1042098 | 2 | uncommon |
| African paradise Monarch | Terpsiphone viridis | 92 | 2.116402116 | 3 | frequent |
| African spoon bill | Platalea alba | 19 | 0.437083046 | 2 | uncommon |
| African thrush | Turdus pelios | 52 | 1.196227283 | 2 | uncommon |
| African wattled lapwing | Vanellus senegallus | 91 | 2.093397746 | 2 | uncommon |
| Black billed wood dove | Turtur abyssinicus | 81 | 1.863354037 | 2 | uncommon |
| Black billed wood hoopoe | Phoeniculus somaliensis | 85 | 1.955371521 | 2 | uncommon |
| Black crake | Amaurornis flavirostra | 7 | 0.161030596 | 2 | uncommon |
| Black headed heron | Areda melanocephala | 27 | 0.621118012 | 2 | uncommon |
| Black headed weaver | Ploceus cucullatus | 197 | 4.531861054 | 3 | frequent |
| Black winged love bird | Agapornis taranta | 54 | 1.242236025 | 2 | uncommon |
| Cardinal woodpecker | Dendropicos fuscescens | 17 | 0.391074304 | 2 | uncommon |
| Cattle egret | Bubulcus ibis | 126 | 2.898550725 | 3 | frequent |
| Common bulbul | Pycnonotus barbatus | 44 | 1.012192317 | 2 | uncommon |
| Common fiscal | Lanius collaris | 33 | 0.759144237 | 2 | uncommon |
| Dark chanting goshawk | Melierax metabates | 25 | 0.575109271 | 2 | uncommon |
| Eastern grey plantain eater | Crinifer zonurus | 84 | 1.93236715 | 2 | uncommon |
| Egyptian goose | Alopochen aegyptiaca | 70 | 1.610305958 | 2 | uncommon |
| Giant kingfisher | Megaceryle maxima | 14 | 0.322061192 | 2 | uncommon |
| Glossy ibis | Plegadis falcinellus | 53 | 1.219231654 | 2 | uncommon |
| Great white egret | Ardea alba | 32 | 0.736139867 | 2 | uncommon |
| Great white pelican | Pelecanus onocrotalus | 189 | 4.347826087 | 3 | frequent |
| Greater blue eared glossy starling | Lamprotornis chalbeus | 267 | 6.142167012 | 3 | frequent |
| Grey headed kingfisher | Halcyon leucocephala | 26 | 0.598113642 | 2 | uncommon |
| Grey headed sparrow | Passer griseus | 81 | 1.863354037 | 2 | uncommon |
| Grey headed woodpecker | Picus canus | 28 | 0.644122383 | 2 | uncommon |
| Grey heron | Ardea cinerea | 16 | 0.368069933 | 2 | uncommon |
| Hadada ibis | Bostrychia hagedash | 70 | 1.610305958 | 2 | uncommon |
| Hammer kop | Scopus umbrett | 62 | 1.426270991 | 2 | uncommon |
| Helmeted Guinea fowl | Numida meleagris | 78 | 1.794340925 | 2 | uncommon |
| Hooded vulture | Necrosyrtes monachus | 34 | 0.782148608 | 2 | uncommon |
| Knob billed duck | Sarkidiornis melanotos | 118 | 2.714515758 | 3 | frequent |
| Laughing dove | Spilopelia senegalensis | 129 | 2.967563837 | 3 | frequent |
| Lemon dove | Columba larvata | 42 | 0.966183575 | 2 | uncommon |
| Little egret | Egretta garzetta | 18 | 0.414078675 | 2 | uncommon |
| Long crested eagle | Lophaetus occipitalis | 34 | 0.782148608 | 2 | uncommon |
| Marabou stork | Leptoptilos crumenifer | 20 | 0.460087417 | 2 | uncommon |
| Namaqua dove | Oena capensis | 40 | 0.920174833 | 2 | uncommon |
| Nubian woodpecker | Campethera nubica | 40 | 0.920174833 | 2 | uncommon |
| Olive thrush | Turdus olivaceus | 77 | 1.771336554 | 2 | uncommon |
| Pied crow | Corvus albus | 74 | 1.702323441 | 2 | uncommon |
| Pin tailed whydah | Vidua macroura | 16 | 0.368069933 | 2 | uncommon |
| Red billed firefinch | *Lagonosticta senegala* | 79 | 1.817345296 | 2 | uncommon |
| Red eyed dove | Streptopelia semitorquata | 218 | 5.014952841 | 3 | frequent |
| Rueppell’s robin chat | Cossypha semirufa | 19 | 0.437083046 | 2 | uncommon |
| Ruppell’s weaver | Ploceus galbula | 87 | 2.001380262 | 2 | uncommon |
| Silvery checked horn bill | Bycanistes brevis | 34 | 0.782148608 | 2 | uncommon |
| Speckled mouse bird | Colius striatus | 36 | 0.82815735 | 2 | uncommon |
| Speckled pigeon | Columba guinea | 365 | 8.396595353 | 3 | frequent |
| Spur winged goose | Plectropterus gambensis | 88 | 2.024384633 | 2 | uncommon |
| Spur winged lapwing | Vanellus spinosus | 37 | 0.851161721 | 2 | uncommon |
| Striped kingfisher | Halcyon chelicuti | 16 | 0.368069933 | 2 | uncommon |
| Swainsan’s sparrow | Passer swainsonii | 17 | 0.391074304 | 2 | uncommon |
| Tawny eagle | Aquila rapax | 18 | 0.414078675 | 2 | uncommon |
| Tropical boubou | Laniarius major | 38 | 0.874166092 | 2 | uncommon |
| Village indigobird | Vidua chalybeata | 19 | 0.437083046 | 2 | uncommon |
| White faced whistling duck | Dendrocygna viduata | 92 | 2.116402116 | 2 | uncommon |
| Yellow billed egret | Ardea intermedia | 25 | 0.575109271 | 2 | uncommon |
| Yellow billed kite | Milvus aegyptius | 22 | 0.506096158 | 2 | uncommon |
| Yellow wagtail | Motacilla flava | 58 | 1.334253508 | 2 | uncommon |

**Suplemetal Table 3: Relative abundance of birds during wet season**

| \| **Common name** \| **Scientific name** \| **No of individual species wet** \| **Wet relative abundance** \| **Abundance score** \| **Abundance Category** \| \| --- \| --- \| --- \| --- \| --- \| --- \| \| Abdim's stork \| *Ciconia abdimii* \| 125 \| 2.23015165 \| 3 \| frequent \| \| Abyssinian ground horn bill \| *Bucorvus abyssinicus* \| 16 \| 0.285459411 \| 2 \| uncommon \| \| Africa Sacrred ibis \| *Threskiornis aethiopicus* \| 114 \| 2.033898305 \| 2 \| uncommon \| \| African black duck \| *Anas sparsa* \| 38 \| 0.677966102 \| 2 \| uncommon \| \| African black headed oriole \| *Oriolus laroatus* \| 79 \| 1.409455843 \| 2 \| uncommon \| \| African darter \| *Anhinga rufa* \| 52 \| 0.927743087 \| 2 \| uncommon \| \| African fish eagle \| *Haliaeetus vocifer* \| 22 \| 0.39250669 \| 2 \| uncommon \| \| African grey horn bill \| *Lophoceros nasutus* \| 16 \| 0.285459411 \| 2 \| uncommon \| \| African hoopoe \| *Upupa africana* \| 15 \| 0.267618198 \| 2 \| uncommon \| \| African jacana \| *Actophilornis africanus* \| 66 \| 1.177520071 \| 2 \| uncommon \| \| African mourning dove \| *Streptopelia decipiens* \| 102 \| 1.819803747 \| 3 \| frequent \| \| African open billed stork \| *Anastomus lamelligerus* \| 68 \| 1.213202498 \| 2 \| uncommon \| \| African paradise Monarch \| *Terpsiphone viridis* \| 88 \| 1.570026762 \| 2 \| uncommon \| \| African spoon bill \| *Platalea alba* \| 26 \| 0.463871543 \| 2 \| uncommon \| \| African thrush \| *Turdus pelios* \| 44 \| 0.785013381 \| 2 \| uncommon \| \| African wattled lapwing \| *Vanellus senegallus* \| 68 \| 1.213202498 \| 2 \| uncommon \| \| Black billed barbet \| *Lybius guifsobalito* \| 28 \| 0.49955397 \| 2 \| uncommon \| \| Black billed wood dove \| *Turtur abyssinicus* \| 98 \| 1.748438894 \| 2 \| uncommon \| \| Black billed wood hoopoe \| *Phoeniculus somaliensis* \| 79 \| 1.409455843 \| 3 \| frequent \| \| Black crake \| *Amaurornis flavirostra* \| 31 \| 0.553077609 \| 2 \| uncommon \| \| Black headed heron \| *Areda melanocephala* \| 33 \| 0.588760036 \| 2 \| uncommon \| \| Black headed weaver \| *Ploceus melanocephalus* \| 252 \| 4.495985727 \| 3 \| frequent \| \| Black winged love bird \| *Agapornis taranta* \| 57 \| 1.016949153 \| 2 \| uncommon \| \| Cardinal woodpecker \| *Dendropicos fuscescens* \| 39 \| 0.695807315 \| 2 \| uncommon \| \| Cattle egret \| *Bubulcus ibis* \| 148 \| 2.640499554 \| 2 \| uncommon \| \| Common bulbul \| *Pycnonotus barbatus* \| 36 \| 0.642283675 \| 2 \| uncommon \| \| Common fiscal \| *Lanius collaris* \| 46 \| 0.820695807 \| 2 \| uncommon \| \| Common Sand piper \| Actitis hypoleucos \| 45 \| 0.802854594 \| 2 \| uncommon \| \| Dark chanting goshawk \| *Melierax metabates* \| 52 \| 0.927743087 \| 2 \| uncommon \| \| Double toothed barbet \| *Lybius bidentatus* \| 58 \| 1.034790366 \| 2 \| uncommon \| \| Eastern Grey plantain eater \| *Crinifer zonurus* \| 121 \| 2.158786798 \| 3 \| frequent \| \| Egyptian goose \| *Alopochen aegyptiaca* \| 90 \| 1.605709188 \| 3 \| frequent \| \| Giant kingfisher \| *Megaceryle maxima* \| 10 \| 0.178412132 \| 2 \| uncommon \| \| Glossy ibis \| *Plegadis falcinellus* \| 42 \| 0.749330955 \| 2 \| uncommon \| \| Great white egret \| *Ardea alba* \| 61 \| 1.088314005 \| 2 \| uncommon \| \| Great white pelican \| *Pelecanus onocrotalus* \| 202 \| 3.603925067 \| 3 \| frequent \| \| Greater blue eared glossy starling \| *Lamprotornis chalbeus* \| 186 \| 3.318465656 \| 3 \| frequent \| \| Grey woodpecker \| *Dendropicos goertae* \| 36 \| 0.642283675 \| 2 \| uncommon \| \| Grey headed sparrow \| *Passer griseus* \| 134 \| 2.390722569 \| 2 \| uncommon \| \| Grey heron \| *Ardea cinerea* \| 25 \| 0.44603033 \| 2 \| uncommon \| \| Hadada ibis \| *Bostrychia hagedash* \| 175 \| 3.12221231 \| 3 \| frequent \| \| Hammer kop \| *Scopus umbrett* \| 88 \| 1.570026762 \| 2 \| uncommon \| \| Helmeted Guinea fowl \| *Numida meleagris* \| 92 \| 1.641391615 \| 2 \| uncommon \| \| Hooded vulture \| *Necrosyrtes monachus* \| 52 \| 0.927743087 \| 2 \| uncommon \| \| Knob billed duck \| *Sarkidiornis melanotos* \| 130 \| 2.319357716 \| 2 \| uncommon \| \| Laughing dove \| *Spilopelia senegalensis* \| 114 \| 2.033898305 \| 2 \| uncommon \| \| Lemon dove \| *Columba larvata* \| 36 \| 0.642283675 \| 2 \| uncommon \| \| Little egret \| *Egretta garzetta* \| 26 \| 0.463871543 \| 2 \| uncommon \| \| Long crested eagle \| *Lophaetus occipitalis* \| 18 \| 0.321141838 \| 2 \| uncommon \| \| Marabou stork \| *Leptoptilos crumenifer* \| 68 \| 1.213202498 \| 2 \| uncommon \| \| Namaqua dove \| *Oena capensis* \| 33 \| 0.588760036 \| 2 \| uncommon \| \| Northern black fly catcher \| *Melaenornis edolioides* \| 74 \| 1.320249777 \| 2 \| uncommon \| \| Nubian woodpecker \| *Campethera nubica* \| 75 \| 1.33809099 \| 3 \| frequent \| \| Olive thrush \| *Turdus olivaceus* \| 89 \| 1.587867975 \| 2 \| uncommon \| \| Pied crow \| *Corvus albus* \| 96 \| 1.712756467 \| 2 \| uncommon \| \| Pin tailed whydah \| *Vidua macroura* \| 28 \| 0.49955397 \| 2 \| uncommon \| \| Red billed firefinch \| *Lagonosticta senegala* \| 23 \| 0.410347904 \| 2 \| uncommon \| \| Red billed oxpeckier \| *Buphagus erythrorhynchus* \| 68 \| 1.213202498 \| 3 \| frequent \| \| Red cheeked cordon blue \| *Uraeginthus bengalus* \| 73 \| 1.302408564 \| 2 \| uncommon \| \| Red eyed dove \| *Streptopelia semitorquata* \| 164 \| 2.925958965 \| 2 \| uncommon \| \| Rueppell’s robin chat \| *Cossypha semirufa* \| 36 \| 0.642283675 \| 2 \| uncommon \| \| Ruppell’s weaver \| *Ploceus galbula* \| 109 \| 1.944692239 \| 2 \| uncommon \| \| Senegal thick knee \| Burhinus senegalensis \| 33 \| 0.588760036 \| 2 \| uncommon \| \| Silvery checked horn bill \| *Bycanistes brevis* \| 58 \| 1.034790366 \| 2 \| uncommon \| \| Speckled mouse bird \| *Colius striatus* \| 22 \| 0.39250669 \| 2 \| uncommon \| \| Speckled pigeon \| *Columba guinea* \| 281 \| 5.01338091 \| 3 \| frequent \| \| Spur winged goose \| *Plectropterus gambensis* \| 62 \| 1.106155219 \| 2 \| uncommon \| \| Spur winged lapwing \| *Vanellus spinosus* \| 29 \| 0.517395183 \| 2 \| uncommon \| \| Striped kingfisher \| *Halcyon chelicuti* \| 48 \| 0.856378234 \| 2 \| uncommon \| \| Swainsan’s sparrow \| *Passer swainsonii* \| 25 \| 0.44603033 \| 2 \| uncommon \| \| Tawny eagle \| *Aquila rapax* \| 27 \| 0.481712756 \| 2 \| uncommon \| \| Tropical boubou \| *Laniarius major* \| 58 \| 1.034790366 \| 2 \| uncommon \| \| Village indigobird \| *Vidua chalybeata* \| 36 \| 0.642283675 \| 2 \| uncommon \| \| Wattled ibis \| *Bostrychia carunculata* \| 98 \| 1.748438894 \| 2 \| uncommon \| \| White faced whistling duck \| *Dendrocygna viduata* \| 99 \| 1.766280107 \| 2 \| uncommon \| \| Yellow billed egret \| *Ardea intermedia* \| 36 \| 0.642283675 \| 2 \| uncommon \| \| Yellow billed kite \| *Milvus aegyptius* \| 51 \| 0.909901873 \| 2 \| uncommon \| \| Yellow fronted canary \| *Crithagra mozambica* \| 101 \| 1.801962533 \| 2 \| uncommon \| \| Yellow wagtail \| *Motacilla flava* \| 26 \| 0.463871543 \| 2 \| uncommon \| |  |  |  |  |
| --- | --- | --- | --- | --- | --- | --- | --- | --- | --- | --- | --- | --- | --- | --- | --- | --- | --- | --- | --- | --- | --- | --- | --- | --- | --- | --- | --- | --- | --- | --- | --- | --- | --- | --- | --- | --- | --- | --- | --- | --- | --- | --- | --- | --- | --- | --- | --- | --- | --- | --- | --- | --- | --- | --- | --- | --- | --- | --- | --- | --- | --- | --- | --- | --- | --- | --- | --- | --- | --- | --- | --- | --- | --- | --- | --- | --- | --- | --- | --- | --- | --- | --- | --- | --- | --- | --- | --- | --- | --- | --- | --- | --- | --- | --- | --- | --- | --- | --- | --- | --- | --- | --- | --- | --- | --- | --- | --- | --- | --- | --- | --- | --- | --- | --- | --- | --- | --- | --- | --- | --- | --- | --- | --- | --- | --- | --- | --- | --- | --- | --- | --- | --- | --- | --- | --- | --- | --- | --- | --- | --- | --- | --- | --- | --- | --- | --- | --- | --- | --- | --- | --- | --- | --- | --- | --- | --- | --- | --- | --- | --- | --- | --- | --- | --- | --- | --- | --- | --- | --- | --- | --- | --- | --- | --- | --- | --- | --- | --- | --- | --- | --- | --- | --- | --- | --- | --- | --- | --- | --- | --- | --- | --- | --- | --- | --- | --- | --- | --- | --- | --- | --- | --- | --- | --- | --- | --- | --- | --- | --- | --- | --- | --- | --- | --- | --- | --- | --- | --- | --- | --- | --- | --- | --- | --- | --- | --- | --- | --- | --- | --- | --- | --- | --- | --- | --- | --- | --- | --- | --- | --- | --- | --- | --- | --- | --- | --- | --- | --- | --- | --- | --- | --- | --- | --- | --- | --- | --- | --- | --- | --- | --- | --- | --- | --- | --- | --- | --- | --- | --- | --- | --- | --- | --- | --- | --- | --- | --- | --- | --- | --- | --- | --- | --- | --- | --- | --- | --- | --- | --- | --- | --- | --- | --- | --- | --- | --- | --- | --- | --- | --- | --- | --- | --- | --- | --- | --- | --- | --- | --- | --- | --- | --- | --- | --- | --- | --- | --- | --- | --- | --- | --- | --- | --- | --- | --- | --- | --- | --- | --- | --- | --- | --- | --- | --- | --- | --- | --- | --- | --- | --- | --- | --- | --- | --- | --- | --- | --- | --- | --- | --- | --- | --- | --- | --- | --- | --- | --- | --- | --- | --- | --- | --- | --- | --- | --- | --- | --- | --- | --- | --- | --- | --- | --- | --- | --- | --- | --- | --- | --- | --- | --- | --- | --- | --- | --- | --- | --- | --- | --- | --- | --- | --- | --- | --- | --- | --- | --- | --- | --- | --- | --- | --- | --- | --- | --- | --- | --- | --- | --- | --- | --- | --- | --- | --- | --- | --- | --- | --- | --- | --- | --- | --- | --- | --- | --- | --- | --- | --- | --- | --- | --- | --- | --- | --- | --- | --- | --- | --- | --- | --- | --- | --- | --- | --- | --- | --- | --- | --- | --- | --- | --- | --- | --- | --- | --- | --- | --- | --- | --- | --- | --- | --- | --- | --- | --- | --- | --- | --- | --- | --- | --- | --- | --- | --- | --- | --- | --- | --- | --- | --- | --- | --- | --- | --- |
